# No Consistent Evidence for Brain Volumetric Correlates of Resilience in Two Independent Cohort Studies

**DOI:** 10.1192/j.eurpsy.2022.549

**Published:** 2022-09-01

**Authors:** A. Cortes Hidalgo, H. Tiemeier, M. Bakermans‑Kranenburg, T. White, T. Banaschewski, M. Van Ijzendoorn, N. Holz

**Affiliations:** 1 Erasmus MC, Child And Adolescent Psychiatry And Psychology, Rotterdam, Netherlands; 2 Vrije Universiteit Amsterdam, Department Of Clinical Child And Family Studies, Amsterdam, Netherlands; 3 Central Institute of Mental Health, Department Of Child And Adolescent Psychiatry And Psychotherapy, Mannheim, Germany; 4 Erasmus University Rotterdam, Department Of Psychology, Education And Child Studies, Rotterdam, Netherlands

**Keywords:** Brain morphology, resilience, adversity, magnetic resonance imaging

## Abstract

**Introduction:**

Childhood adversities have been associated with long-lasting brain morphological differences and poor psychological outcomes over the lifespan. Evidence with regard to protective factors counteracting the detrimental effect of childhood adversity on neurobiology is scarce.

**Objectives:**

Therefore, we examined the interplay of childhood adversity with multiple protective factors in relation to brain morphology in a child and an adult cohort.

**Methods:**

We analyzed data from two epidemiological longitudinal birth cohorts, the Generation R Study (N=3,008) and the Mannheim Study of Children at Risk (MARS) (N=179). Cumulative exposure to 12 adverse events (such as physical and sexual abuse), and the presence of protective factors, including child temperament, cognition, self-esteem, friendship quality and maternal sensitivity were assessed at different time points during childhood. Anatomical scans were acquired at the ages of 9-11 years in Generation R and at 25 years in MARS.

**Results:**

Childhood adversity was related to smaller global brain volumes in Generation R, with similar effect sizes observed for the cerebellar volume in MARS. While small interaction effects between adversity and protective factors were found on the medial orbitofrontal cortex, the cerebellum and the amygdala in either cohort study, no interactions were consistent across cohorts or survived correction for multiple comparisons.

**Conclusions:**

We found no consistent or strong evidence for interaction effects between multiple protective factors and childhood adversities on brain structure in a child and an adult cohort study. Instead, small interaction effects were found in either children or adults warranting further investigation and more fine-grained analyses.

**Disclosure:**

TB:consultancy for Actelion, Hexal Pharma, Lilly, Lundbeck, Medice, Novartis, Shire; conference support by Lilly, Medice, Novartis, Shire; clinical trials by Shire and Viforpharma; royalties by Hogrefe, Kohlhammer, CIP Medien, Oxford University Press

